# Editorial: Association between diabetic nephropathy and diabetic retinopathy or non-diabetic nephropathy

**DOI:** 10.3389/fendo.2024.1359011

**Published:** 2024-01-25

**Authors:** Haiyong Chen, Xuefei Tian, Xiaoyong Yu

**Affiliations:** ^1^ School of Chinese Medicine, Li Ka Shing Faculty of Medicine, The University of Hong Kong, Hong Kong, Hong Kong SAR, China; ^2^ Department of Chinese Medicine, The University of Hong Kong-Shenzhen Hospital, Shenzhen, China; ^3^ Section of Nephrology, Department of Internal Medicine, Yale University School of Medicine, New Haven, CT, United States; ^4^ Department of Nephrology, Shaanxi Provincial Hospital of Traditional Chinese Medicine, Xi’an, China

**Keywords:** diabetic retinopathy, non-diabetic nephropathy, diabetic nephropathy, Klotho pathway, ferroptosis, Mendelian randomization (MR) analysis

Diabetes is a chronic metabolic disorder characterized by hyperglycemia, which can lead to severe complications if not properly managed. Among these complications, diabetic nephropathy (DN) and diabetic retinopathy (DR) are two common and serious complications that significantly impact the quality of life for those with diabetes. Non-diabetic nephropathy (non-DN), on the other hand, refers to kidney damage that occurs in individuals without diabetes. This topic aims to explore the intricate association between DN and both DR and non-DN.

DN is a leading cause of end-stage kidney disease worldwide, occurring when high blood glucose levels damages the blood vessels and tissues in the kidney. DR is a complication of diabetes that can cause damage to the blood vessels in the retina leading to vision problems and possible blindness if left untreated. Both conditions are believed to share common risk factors and underlying mechanisms, while the causal relationship remains elusive until Fang et al. utilized 20 genetic variants, associated with DR. Their Mendelian randomization method provided evidence of a causal link between DN and DR, reinforcing the idea of shared underlying mechanisms.

Tang et al.‘s study suggested the klotho pathway might be the common pathological mechanism for the development of DN and DR as the disfunction of klotho is associated with inflammation, fibrosis, oxidative stress, and dis-regulation of calcium and phosphate hemostasis, in both DN and DR.

Ferroptosis is a recently discovered type of cell death that is identified by the accumulation of lipid peroxides to deadly levels, which is dependent on iron (Li et al.). Patients with DN often experience iron metabolic disorders. In their article, Li et al. emphasize that ferroptosis serves as a common pathological mechanism in both DN and DR. They highlighted the potential therapeutic implications of targeting ferroptosis pathways for the prevention and treatment of DN and DR.

In the Research Topic, several potential risk factors for the development of DN were studied using the Mendelian randomization approach. Ren et al. found that the reduction of lean mass in muscle (sarcopenia) has the higher risk of DN onset, and the DN onset also decreases grip strength, but overall, no causal relationship was identified between sarcopenia and DN. Another study investigated the relationship between coffee consumption and the risk of DN (Fang et al.). The findings of this study indicated a potential protective effect of coffee intake against DN (Fang et al.).

Zhang et al. evaluated the association of serum amino acids involved in the urea cycle with the risk of chronic kidney disease (CKD) in patients with Type 2 diabetes mellitus (T2DM). They found that higher serum citrulline and a lower ratio of ornithine/citrulline could predict the risk of CKD. Furthermore, another study conducted a systematic review and meta-analysis to analyze the prediction value of functional magnetic resonance imaging (fMRI) parameters on DN among patients and health controls. They identified a few specific parameters for determining the early onset of DN (Zhang et al.).

These studies have shed light on the association between DN and DR, suggesting the shared causal relationship and common underlying mechanisms, e.g., the involvement of Klotho and ferroptosis pathways. These studies also have found the potential protective effect of coffee intake for DN, potential serum biomarkers, and fMRI in DN diagnosis. However, causal relationships between sarcopenia and DN were not confirmed in this study.

While the association between DN and DR has been extensively studied, further research is warranted to explore the connection between DN and non-DN. Understanding the association between these two conditions is crucial for accurate diagnosis and effective management. Promisingly, fMRI has shown potential in evaluating diabetic kidney disease, aiding in early detection and treatment.

In conclusion, a better understanding of the intricate association between DN, DR, and non-DN is paramount for developing effective prevention strategies and treatment approaches. Further research, utilizing methodologies such as Mendelian randomization, will continue to provide valuable insights into the underlying mechanisms and potential therapeutic targets for these diabetes-related complications.

## Author contributions

HC: Writing – original draft, Writing – review & editing, Conceptualization. XT: Writing – original draft, Writing – review & editing, Conceptualization. XY: Writing – original draft, Writing – review & editing, Conceptualization.

